# Editorial: Environmental phytoremediation: plants and microorganisms at work

**DOI:** 10.3389/fpls.2015.00520

**Published:** 2015-07-07

**Authors:** Antonella Furini, Anna Manara, Giovanni DalCorso

**Affiliations:** Department of Biotechnology, University of VeronaVerona, Italy

**Keywords:** phytoremediation, plant-microbe interaction, ecopiling, metal hyperaccumulator, endophytic bacteria

Human industry, farming, and waste disposal practices have resulted in the large-scale contamination of soil and water with organic compounds and heavy metals, with detrimental effects on ecosystems and human health. Conventional soil remediation methods are expensive and often involve the storage of soil in designated areas, postponing rather than solving the problem. In the last decade, the pressing need to find alternative methods has highlighted the scientific and economic benefits of plants and their associated microorganisms, which can be used for the reclamation of polluted soil and water (Meagher, 2000). This is an elegant and low-cost approach for the decontamination of polluted sites and has been greeted with a high degree of public acceptance, therefore prompting research into the use of phytoremediation technology to address the large areas of land and water currently affected (reviewed by Krämer, 2005; Vangronsveld et al., 2009; Lee, 2013). This *Frontiers in Plant Science* research topic provides a snapshot of current research into the application of environmental phytoremediation strategies.

Many scientists are currently investigating the phenomenon of metal hyperaccumulation in different species, aiming to determine the mechanisms associated with the accumulation and detoxification of heavy metals and ultimately to use these plants and their rhizosphere-derived microorganisms for the decontamination of polluted sites. A greenhouse experiment using *Pteris vittata* with or without bacterial strains selected from autochthonous rhizosphere-derived microorganisms [chosen for their resistance to high concentrations of arsenic (As) and their ability to reduce arsenate to arsenite] showed that the efficiency of phytoextraction increased when *P. vittata* plants were inoculated with the selected microbial communities (Lampis et al., 2015). A detailed comparative analysis of the endophytic bacteria and fungi from the selenium (Se) hyperaccumulator species *Stanleya pinnata* (Brassicaceae) and *Astragalus bisulcatus* (Fabaceae), and the related non-accumulators *Physaria bellis* (Brassicaceae) and *Medicago sativa* (Fabaceae), revealed that isolates from Se hyperaccumulator species were more resistant to selenate and selenite, could reduce selenite to elemental Se, could reduce nitrite and produce siderophores, and several strains also showed the ability to promote plant growth (Jong et al., 2015). Microorganisms with high Se tolerance and the ability to produce elemental Se would be useful for wastewater treatment and/or the production of Se nanoparticles (Staicu et al., 2015).

The use of omics analysis and advanced microscopy to study the interaction between metal hyperaccumulators and the bacterial rhizobiome is considered in a review article by Visioli et al. (2015). This emphasizes emerging techniques for the analysis of microbial communities in polluted soils that help to determine the impact of pollution on those communities (Berg et al., 2012). It also highlights the advantages of *in situ* analysis to monitor the colonization of plants and the survival of microbial inoculums under real conditions, particularly the use of environmental scanning electron microscopy, a powerful approach for the *in situ* analysis of biological specimens without sample preparation (Stabentheiner et al., 2010; Visioli et al., 2014).

The phytoremediation potential of plants inoculated with bacteria isolated from the rhizosphere and endosphere of other plants grown in soil contaminated with heavy metals is discussed in two articles (Khan et al., 2015; Ma et al., 2015). The arboreal species *Prosopis juliflora*, native to South America, was previously considered as a bioindicator species for polluted sites (Senthilkumar et al., 2005) and was shown to tolerate high concentrations of heavy metals and therefore may be useful in soil reclamation (Varun et al., 2011). Several bacterial strains with resistance to chromium (Cr), isolated from the rhizosphere and endosphere of *P. juliflora* plants grown on soil contaminated with tannery effluent, also showed tolerance toward other heavy metals such as Cd, Cu, Pb, and Zn. The inoculation of ryegrass (*Lolium multiflorum* L.) with three of these isolates promoted plant growth and the removal of toxic metals from polluted soil, demonstrating that the interaction between plants and bacterial strains identified in contaminated areas could improve plant growth and the efficiency of phytoremediation (Khan et al., 2015). Likewise, *Brassica juncea* and *Ricinus communis* plants inoculated with rhizospheric and endophytic bacteria isolated from a polluted serpentine environment accumulated more biomass and heavy metals than non-inoculated control plants (Ma et al., 2015). These effects were clearly attributed to the production of bacterial metabolites that promoted plant growth and metal mobilization. However, the low metal translocation factor obtained upon inoculation indicated that metal-resistant serpentine bacteria are suitable for the phytostabilization of contaminated soil (Ma et al., 2015).

The beneficial interaction between plants and rhizobia for the remediation of contaminated soil is discussed by Teng et al. (2015). Certain symbiotic relationships between legumes and nitrogen-fixing bacteria are resistant to heavy metals, promoting the dissipation of organic pollutants and enhancing their removal (Fan et al., 2008; Glick, 2010; Li et al., 2013). Rhizobia not only fix nitrogen but also promote plant growth, thus increasing plant biomass, soil fertility, the bioavailability, uptake and translocation of pollutants, the degradation of organic contaminants and the phytostabilization of metals. All these features make rhizobia valuable phytoremediation tools. Endophytic rhizobia degrade organic contaminants that have accumulated in nodules, thus reducing phytovolatilization and facilitating phytoremediation in polluted environments (Teng et al., 2015).

Two further articles discuss the use of plants and their associated microorganisms for the reclamation of land polluted with organic contaminants (Germaine et al., 2015; Sauvêtre and Schröder, 2015). In the first project (Sauvêtre and Schröder, 2015), *Phragmites australis* plants were exposed to carbamazepine, a widely-used drug that is present in the environment as a persistent and recalcitrant contaminant (Ternes et al., 2007; Huerta-Fontela et al., 2011). After 9 days, the plants reduced the initial drug concentration by 90%, and characterization of the endophytic bacteria revealed that all isolates possessed at least one plant growth-promoting trait. Several had the ability to remove carbamazepine from soil, whereas others produced siderophores and were able to solubilize phosphate, suggesting they would be beneficial in phytoremediation programs. The second article addresses the effectiveness of a large-scale combined phytoremediation/biopiling system, termed ecopiling, for the removal of hydrocarbons from soil affected by industrial contamination (Germaine et al., 2015). Bacterial communities capable of total petroleum hydrocarbon (TPH) degradation were used to inoculate soil contaminated with chemical fertilizers. Perennial rye grass and white clover were then sown to complete the ecopile. During a 2-year trial, there was a consistent reduction in the TPH level suggesting that this multifactorial approach involving biostimulation, bio-augmentation and phytoremediation is suitable for the remediation soils contaminated with industrial hydrocarbons.

It is notable that all the articles submitted in this research topic focused on the use of naturally-occurring hyperaccumulator species rather than transgenic plants and/or microorganisms, although genetically-engineered plants and microbes can also be used for the efficient treatment of polluted soil and water (Van Aben, 2009; Singh et al., 2011). This highlights the diverse and promising approaches that are being developed by the environmental phytoremediation research community.

## Conflict of interest statement

The authors declare that the research was conducted in the absence of any commercial or financial relationships that could be construed as a potential conflict of interest.
